# Comparison of Diagnostic Validity of Cephalometric Analyses of the ANB Angle and Tau Angle for Assessment of the Sagittal Relationship of Jaw and Mandible

**DOI:** 10.3390/jcm12196333

**Published:** 2023-10-02

**Authors:** Jacek Kotuła, Anna Kuc, Ewa Szeląg, Alicja Babczyńska, Joanna Lis, Jacek Matys, Beata Kawala, Michał Sarul

**Affiliations:** 1 Department of Dentofacial Orthopedics and Orthodontics, Wroclaw Medical University, Krakowska 26, 50-425 Wroclaw, Poland; dental.star@wp.pl (A.K.); ewa.szelag@umw.edu.pl (E.S.); ortodoncja@umw.edu.pl (A.B.); joanna.lis@umw.edu.pl (J.L.); beata.kawala@umw.edu.pl (B.K.); michal.sarul@umw.edu.pl (M.S.); 2 Oral Surgery Department, Wroclaw Medical University, Krakowska 26, 50-425 Wroclaw, Poland; 3 Department of Orthodontics, Technische Universitat Dresden, 01307 Dresden, Germany

**Keywords:** cephalometric analyses, diagnosis, orthodontics

## Abstract

Background: Cephalometric analysis is an essential tool used in orthodontic diagnosis and treatment planning. The aim of this study was to evaluate the reliability and repeatability of new cephalometric points introduced in Tau angle analysis, in contrast to the gold standard, which is the analysis of the ANB angle. For this purpose, an attempt was made to assess the repeatability and reliability of the introduction of anthropometric points by evaluating both inter- and intraobserver parameters, as well as the agreement among the orthodontists participating in the study. Methods: Repeatability and reliability assessments for all six anthropometric points (N, A, B, T, M, G) used in the analysis of the ANB and Tau angles were conducted individually by 29 orthodontists. This assessment was performed in triplicate on the day of the study, on the day following the first study, and on the seventh day after the second study. Measurement errors for the ANB and Tau angles were evaluated using the Dahlberg formula and intraclass correlation coefficients (ICCs). Results: The orthodontists in the study measured sagittal discrepancy significantly more accurately using the ANB angle compared to the Tau angle (*p* < 0.001). The Dahlberg error for measuring the Tau angle was three times greater than that for the ANB angle (*p* < 0.001). Additionally, the ICC for the Tau angle was more than 3.5 times smaller than that for the ANB angle, while the R&R error for Tau measurement was more than three times greater than that for the ANB angle (*p* < 0.001). Conclusions: The results of ANB angle measurements exhibit fewer errors in comparison to Tau angle measurements.

## 1. Introduction

Orthodontic treatment planning relies on the accurate diagnosis of skeletal discrepancies, necessitating precise cephalometric parameters. Cephalometric analysis serves as a crucial tool in assessing the sagittal dimension and compatibility of mandibular–maxillary bases [1,2]. To ensure the reliability of diagnostic data obtained through cephalometric analysis, it is imperative to maintain stability, clarity, and repeatability in landmark identification, as well as in linear and angular measurements [2].

In 2022, a new systematic review was published, which included new linear and angular measurements to assess the sagittal discrepancy of maxillary bases [1]. Few studies utilizing new methods of cephalometric evaluation have been published in recent years. An example of such an approach is the Tau method developed by Gupta et al. [3]. This angle is determined by three T landmarks (new landmarks), as well as G and M points, which form T–G and G–M auxiliary lines. The Tau angle is measured between them, identifying the mandibular position in relation to the maxilla in the sagittal dimension. The development of this new analysis, used for determining the sagittal position of the maxillary bases in relation to each other, has raised numerous objections related to the stability and repeatability of previous cephalometric parameters. The authors made efforts to address issues with the repeatability of cephalometric parameter analyses, which, among other things, are associated with the following:

1. ANB angle stability due to growth changes and the instability of the position of point N, which affects the size and clarity of the ANB angle during growth [4,5].
2. Changes in the measurement of the length of the skull base, which affects the ANB angle [4,5].
3. Rotation of the condyle at the temporomandibular joint, which influences changes in the sagittal relationship between the maxillary bases in relation to each other and the rotation of the mandible during orthodontic treatment.
4. Wits assessment [5], which is related to the instability of the occlusal plane. It can be challenging to accurately determine the precise occlusal plane at various stages of tooth development and dental age. The irregularity of the occlusal plane is also influenced by factors such as missing teeth, malocclusions, and mandibular deformities.
5. Assessment of the W angle, which is measured between the perpendicular line to point M on the SG line and the MG line [6]. Although W angle analysis utilizes points M and G (the same points used in Tau angle analysis), which are relatively stable and do not undergo relocation due to remodeling associated with tooth movements, the S point, on the other hand, is highly unstable as it moves backward and downward during growth.
6. Imprecision and difficulty of determining the Beta angle [7]. The Beta angle is the angle formed by the perpendicular line drawn from point C to point A, intersecting with the line AB. This angle utilizes three distinctive skeletal elements—point A, point B, and the prominent condylar axis—to measure an angle that indicates the severity and type of skeletal dysplasia in the sagittal dimension [8]. Beta angle analysis relies on point A as a landmark, and changes in its position are associated with alveolar remodeling resulting from orthodontic movements. Additionally, determining the position of the mandibular condyle can be challenging, which consequently limits the reliability of the Beta angle.
7. YEN angle assessment [9] is based on landmarks such as S, M (midpoint of the anterior maxilla), and G (center at the bottom of the symphysis), which together form the YEN angle measured at point M. Points M and G are the same as those used in Tau angle analysis. The imprecision arises from the fact that point G shifts in a manner resembling the letter ‘S’ during growth.

The authors of the Tau angle method consider the location of the center of the maxilla and the mandibular symphysis as precise and appropriate compared to other reference points in the analysis of sagittal discrepancy of the maxillary bases, and as more stable compared to points N and S in the correct assessment of the sagittal discrepancy of the designated T point [3]. The T point is located at the highest point at the junction of the frontal wall of the pituitary fossa and the tuberculum sellae. The M and G points are also stable. Analysis of the Tau values between 28° and 34° suggests skeletal Class I malocclusion; values below 28° indicate skeletal Class III; and those above 34° suggest skeletal Class II [3]. However, as mentioned above, each cephalometric measurement is only of diagnostic value if the points that define it are easily determined in a clear and repeatable manner using a digital image. Moreover, the introduction of a new measurement only makes sense if its use either enables the diagnosis to be made in a clearer, more confident manner or enables other diagnostic benefits to be achieved.

Orthodontic treatment planning is based on the correct diagnosis of skeletal discrepancy, which requires accurate and precise cephalometric parameters. This article aims to assess the measurement accuracy of two cephalometric angles (ANB—the gold standard in the sagittal discrepancy analysis—and the new Tau angle) based on nine cephalograms of patients differing in terms of skeletal class.

The aim of selecting cephalometric images with typical skeletal classes was to assess whether the accuracy and repeatability of determining anthropometric points, and, thus, the results of internal sagittal analysis in all configurations, are comparable, or whether the skeletal discrepancy affects the reliability of the entered points and measurements.

## 2. Materials and Methods

This study was approved by the Bioethical Committee at the District Medical Chamber in Zielona Góra, decision no. 01/173/2023 of 6 March 2023, and informed consent in accordance with the Declaration of Helsinki was obtained from all participating subjects/their parents. The sample size of 29 orthodontists/observers was calculated using G*Power (Kiel University, Germany) software based on preliminary measurements with a significance level of 0.05, d = 0.5, 95% confidence intervals, and 83% power. All subjects were selected to meet the inclusion criteria, representing patients before orthodontic treatment with various Angle Class I, Class II, and Class III categories characterized by different base angles, while also meeting conditions of being free from systemic illnesses, or untreated dental and periodontal disease.

**Figure 1:**
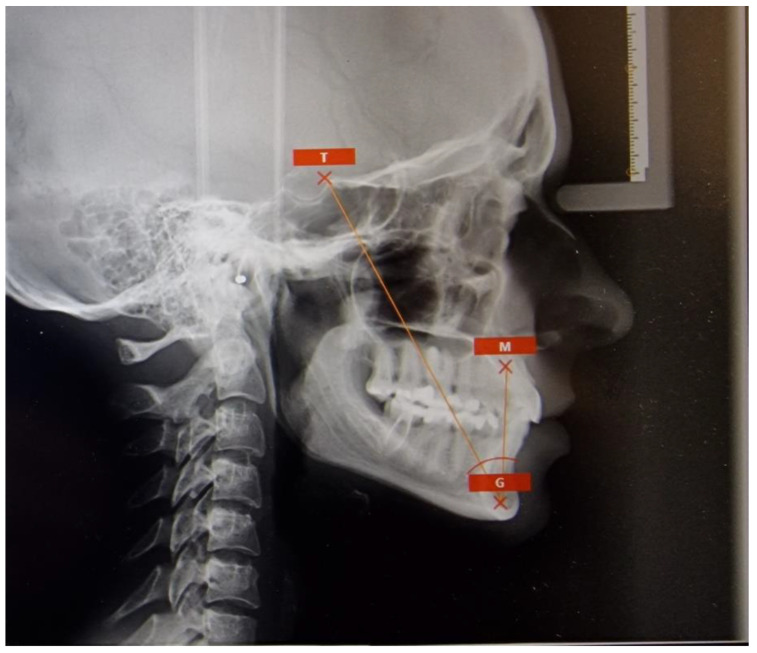
The landmarks T,M,G used in Tau angle analysis.

**Figure 2:**
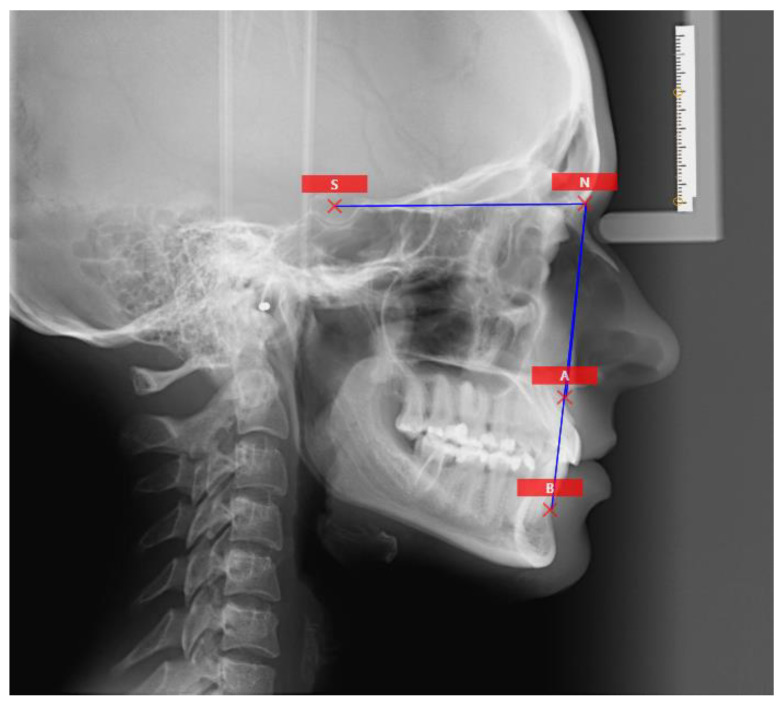
The landmarks used in ANB analysis.

The authors evaluated the reliability of lateral cephalograms and their ANB angle cephalometric analysis in correlation with the newly introduced Tau angle analysis, which was not present in the scientific literature prior to the conclusion of 2021 [1,3] (Figure 1 and Figure 2).

Cephalometric radiographs were taken with the patient’s head correctly fixed in a cephalostat. Oils were inserted centrally into the external ear canals. The correctness of a beam path perpendicular to the sagittal plane was verified. The head was positioned in such a way that the Frankfort horizontal plane was parallel to the floor. Patients were advised to grit their teeth in central occlusion and have a slightly closed mouth. 

The position of the six cephalometric points was determined on 9 radiographs. These were the coordinates of the N, A, B, T, M and G points from which ANB and Tau were determined. The software Orthodontics v. 9 was used for determining the coordinates of the points and calculating the angles. Analyses were performed using NEC Multisync EA 244 WMi (NEC, Tokyo, Japan) medical monitors, certified by the Diagnostic Equipment Quality Laboratory.

Finally, 9 cephalometric radiographs of patients before orthodontic treatment were used for the study:

- Cephalogram 1, showing Angle Class I patient “A” with a high base angle;
- Cephalogram 2, showing Angle Class I patient “D” with an average base angle;
- Cephalogram 3, showing Angle Class I patient “E” with a low base angle;
- Cephalogram 4, showing Angle Class II patient “B” with a high base angle;
- Cephalogram 5, showing Angle Class II patient “F” with an average base angle;
- Cephalogram 6, showing Angle Class II patient “G” with a low base angle;
- Cephalogram 7, showing Angle Class III patient “C” with a high base angle;
- Cephalogram 8, showing Angle Class III patient “H” with an average base angle;
- Cephalogram 9, showing Angle Class III patient “I” with a low base angle.

The assessment of 450 images was performed independently by two authors of this article (J.K., A.E.K.)—only correctly taken cephalograms were used. The authors initially conducted a cephalometric analysis following the Segner Hasund method to categorize the photos into the appropriate groups based on the specified criteria. The following exclusion criteria were used:

1. The presence of asymmetry that is visible on the radiograph and is interpreted as greater than 10% divergence of contours of the right and left mandibular bases;
2. Landmarks on cephalograms that could not be identified due to a projection error or an incorrect contrast;
3. Bilateral anatomical structures that did not overlap properly by superimposing on the mediolateral plane.

From this selected group of cephalograms, one cephalogram was chosen for each of the 9 patient groups mentioned earlier, matching the appropriate Angle Class and angle of the maxillary bases. Twenty-nine orthodontists were invited to participate in the study and received initial training on accurately identifying anthropometric points for both the ANB angle and the Tau angle. Each researcher conducted the ANB and Tau angle analyses three times, on days 0, 1, and 7.

The landmark identification was carried out manually on digital images using a cursor controlled by a computer mouse. The results were recorded in an Excel spreadsheet (Microsoft, Seattle, WA, USA). Subsequently, statistical analysis was conducted. The mean and standard deviation of the Tau angle for each of the 9 cephalograms, related to individual skeletal defects in the sagittal plane, were measured in correlation with the size of the base angles to assess the stability of the Tau angle in evaluating skeletal defects. The obtained values were then correlated with parameters from the ANB angle analysis.

In total, six cephalometric points were identified on each of the 9 radiographs. These points consisted of coordinates for the N, A, B, T, M, and G points, which were used to determine the ANB and Tau angles in patients before orthodontic treatment. This analysis was conducted by a lead researcher and 28 randomly selected observers, all of whom were orthodontists. Digital contours and measurements were collected in triplicate on the day of the study, on the day following the initial study, and on day 7 following the second study. The positions of landmarks determined by the 29 observers were assessed in relation to the x-axis (horizontal divergence) and the y-axis (vertical divergence) as mean values and standard deviations (Figure 1 and Figure 2).

The reliability of the method, signifying the significance of the relationship between the coordinates of the points included in determining cephalometric angles, was assessed using the Pearson correlation coefficient (r^2^). The level of statistical significance was set at *p* < 0.05. The repeatability between methods was calculated by determining errors using Dahlberg’s formula and assessing interclass and intraclass correlation coefficients (ICCs). Intraclass correlation coefficient values were interpreted according to Koo and Li [10]. The evaluation of the repeatability and reproducibility of measurements was based on the variability (variance) of the coordinates of designated points. The assessment was divided into the following three groups:

- Variability in the position of cephalometric points among the group of 9 studied patients (between-group variance);
- Variability in the position of cephalometric points made by 29 different doctors (reproducibility);
- Measurement errors across three measurements made by the same doctor on the same patients (repeatability).

The R & R (Repeatability and Reproducibility) module of STATISTICA v.13.3 (TIBCO Software Inc., Palo Alto, CA, USA) was utilized for the analysis of repeatability and reproducibility. When analyzing ANB and Tau values for assessing the correct classification of patients into one of three skeletal classes, the Cohen’s kappa coefficient was used. Cohen’s kappa is the reliability coefficient for measuring the same variable twice, which is the nominal and dependent variable. Cohen’s kappa takes values from −1 to 1. The closer the value is to 1, the more concordant the assessments are when using the two methods.

## 3. Results

The mean values presented in the analysis were calculated from a comprehensive dataset encompassing a total of 87 measurement results. These results were obtained through the combined measurements of 29 different doctors, each performing three repetitions of measurements for both the ANB and Tau angles. The measurements were conducted using the coordinates associated with points A, N, B, T, G, and M, ensuring a thorough and robust assessment of these cephalometric angles (Table 1).

### 3.1. ANB Angle

The ANB angle is determined by the coordinates of points A, N, and B, and it can be calculated according to the following relationship:

ANB = arc tg[(N_y_ − A_y_)/(N_x_ − A_x_) − (N_y_ − B_y_)/(N_x_ − B_x_)]/[1+ (N_y_ − A_y_)/(N_x_ − A_x_) ∗ (N_y_ − B_y_)/(N_x_ − B_x_)]

Therefore, the accuracy of the ANB angle identification depends on the location accuracy on the cephalograms of points A, N, and B. Table 2 shows the mean values obtained from 87 measurement results (29 doctors × 3 repetitions) for the ANB angle and coordinates of points A, N, and B. The A and B dispersions are vertical (variability is greater in the direction of the vertical axis AΔA_x_), while N dispersion is concentric (variability in both directions is of the same order). The location accuracy of the horizontal coordinates of points A, N, and B is approximately twice that of the vertical coordinates. The accuracy of the ANB angle measurement is most affected by the accuracy of the horizontal location of points A (A_x_ coordinate) and B (B_x_) and both coordinates of point N (Table 3).

The maximum ANB angle is described by coordinates such as A_x_^max^, A_y_^max^, N_x_^avg^, N_y_^min^, B_x_^min^, and B_y_^max^, while the minimum angle is described by coordinates such as A_x_^min^, A_y_^min^, N_x_^avg^, N_y_^max^, B_x_^max^, and B_y_^min^. 

The significance of the relationship between the coordinates of points A, N, and B and the ANB angle was confirmed by the values of the Pearson correlation coefficients. The most significant correlation was for the A_x_ (six patients) and A_y_ (five patients) coordinates (Table 4).

The strong relationship between the ANB angle and the A_x_ variable was also confirmed by the results of the multiple regression analysis (Table 5).

The study participants, consisting of 29 orthodontists, analyzed nine cephalograms in triplicate. The results from the first and third measurements (taken one week apart) were used to assess repeatability. Errors were calculated using Dahlberg’s formula, and interclass and intraclass correlation coefficients (ICCs) were determined. The Dahlberg error ranged from 0.265 to 0.665 (Table 6), while the ICC results ranged from 0.841 to 1.000, indicating a very high correlation between the measurements conducted by the 29 orthodontists (Table 7).

The ICC is used for determining whether the results of cephalometric measurements can be assessed in a reliable way by different orthodontists.

ICC values were interpreted according to Koo and Li [10] as follows:

- <0.50: Poor reliability of assessors;
- 0.5–0.75: Moderate reliability of assessors;
- 0.75–0.9: Good reliability of assessors;
- >0.9: Excellent reliability of assessors.

Consequently, an ICC of 0.782 for the ANB angle indicates that the reliability of the 29 assessing orthodontists can be considered “good”.

The method employed to assess the repeatability and reproducibility of ANB angle measurements is outlined in Table 8. The variability (variance) of the coordinates of the designated points was categorized into three groups:

- Variability in the position of cephalometric points among the group of nine studied patients (between-group variance);
- Variability in the position of cephalometric points made by the 29 different doctors (reproducibility);
- Measurement errors across three measurements made by the same doctor on the same patients (repeatability).

In the ideal scenario, almost all of the variability in measurement results should be attributed to between-group variance (individual patient variability), with only a negligible portion of variability arising from incomplete reproducibility among orthodontists and incomplete repeatability of measurements. The R & R (Repeatability and Reproducibility) module of STATISTICA v.13.3 (TIBCO Software Inc., Palo Alto, CA, USA) was employed to analyze repeatability and reproducibility.

The results for the ANB angle are presented in Table 8. In the last column, values indicating the relative contributions of variability from different sources are provided: repeatability accounts for 1.61%, reproducibility (among different doctors) accounts for 0.92%, interpatient variability accounts for 97.47%, and combined repeatability and reproducibility (R&R) accounts for 2.53%. In conclusion, the vast majority of the variability in the ANB angle is attributed to individual patient differences (cephalograms). This is a positive outcome that supports a favorable evaluation of the ANB angle measurement method. The total R&R result for ANB is 2.53%, well below 10%, signifying satisfactory quality (the highest acceptable R&R value being 15%).

### 3.2. Tau Angle

The Tau angle is a parameter used to determine the true bony sagittal maxillomandibular relationship (Figure 1). The Tau angle is constructed by identifying three cephalometric landmarks: Point T, which is the uppermost point at the junction of the frontal wall of the pituitary fossa and the tuberculum sellae; Point M, a constructed point representing the center of the largest circle that touches the frontal, upper, and palatal surfaces of the maxilla; and Point G, the focal point of the largest circle that touches the inner frontal, posterior, and lower edge of the mandibular symphysis. The Tau angle is formed between the two lines connecting points T and G and points M and G. The objective of the current study was to establish the mean and standard deviation of the Tau angle for three skeletal malocclusions. A Tau angle between 28° and 34° suggests a skeletal Class I malocclusion, while values below 28° indicate a Class III skeletal pattern, and values above 34° suggest a skeletal Class II pattern.

The Tau angle is determined by the coordinates of points T, G, and M and can be calculated according to the following relationship:

Tau = arc tg[(G_y_ − T_y_)/(G_x_ − T_x_) − (G_y_ − M_y_)/(G_x_ − M_x_)]/[1+ (G_y_ − T_y_)/(G_x_ − T_x_) ∗ (G_y_ − M_y_)/(G_x_ − M_x_)]

The accuracy of Tau angle measurement relies on the precise positioning of the three points T, G, and M on the cephalograms. Table 9 displays the mean values of 87 Tau angle measurements, along with the coordinates of points T, G, and M. The dispersion of point G is vertical (with greater variability in the direction of the vertical axis, Δgy/ΔGx > 1), while the dispersions of points T and M are horizontal (Δty/ΔTx and Δmy/ΔMx < 1). The accuracy of Tau angle measurement is most significantly affected by the horizontal positioning accuracy of point M (Mx coordinate) and point T (Tx) (Table 10).

The significance of the relationship between the coordinates of points T, G, and M and the Tau angle was confirmed by the values of the Pearson correlation coefficients (Table 11). The most significant correlation was for the T_x_ and M_x_ coordinates (six patients).

The strong relationship between the ANB angle and the A_x_ variable was also confirmed by the results of the multiple regression analysis (Table 12).

The measurement errors for the angle Tau and the coordinates of points T, B, and M were estimated according to the Dahlberg formula and the ICCs. The Dahlberg error ranged from 0.891 to 1.639 (Table 13), while the results for ICC ranged from 0.147 to 0.624, which indicates a weak correlation between the measurements made by the 29 orthodontists (Table 14).

The results of the analysis of repeatability and reproducibility for the Tau angle are presented in Table 15. The repeatability of the results accounts for 4.30% of the total variability, reproducibility (among different doctors) accounts for 3.94%, interpatient variability accounts for 91.76%, and combined repeatability and reproducibility (R&R) accounts for 8.24%. Consequently, the vast majority of the variability in the Tau angle can be attributed to individual patient differences (cephalograms). This is a positive outcome that supports a favorable evaluation of the Tau angle measurement method. The total R&R result for Tau is 8.24%, which falls below 10%, indicating satisfactory quality.

### 3.3. ANB vs. Tau

The orthodontists involved in the study demonstrated significantly greater accuracy in measuring the ANB angle compared to the Tau angle. The Dahlberg error for Tau angle measurements was approximately three times larger than that for the ANB angle. Similarly, the ICC for the Tau angle was more than three and a half times smaller than that for the ANB angle. Furthermore, the R&R error for Tau angle measurements was more than three times larger compared to that for ANB angle measurements (Table 16). In summary, the results indicate that the ANB angle measurements exhibit less error compared to the Tau angle measurements. This discrepancy is influenced by the smaller dispersion of horizontal coordinates of the points that have the greatest impact on angle measurement error.

The ability to accurately classify patients into one of the three skeletal classes based on the measured ANB and Tau angles is similar (*p* < 0.001). The Cohen’s kappa value based on the ANB angle values and the gold standard is 0.778, which is slightly higher (indicating better agreement) than that based on the Tau angle values (0.722). Cohen’s kappa is a reliability coefficient used to measure the consistency between two methods measuring the same variable, which is nominal and dependent. Cohen’s kappa values range from −1 to 1, with values closer to 1 indicating greater agreement between the two methods (Table 17).

## 4. Discussion

Cephalometric assessment of sagittal discrepancy plays a crucial role in orthodontic evaluation, facilitating the development of appropriate treatment plans [11,12]. Over time, numerous researchers have sought to identify the most stable and reproducible anthropometric points, regardless of the growth direction or orthodontic treatment provided. However, it is important to note that no method is entirely free of errors, and in certain situations, the obtained results may need validation through an alternative method [12]. Most studies focusing on cephalometric analysis typically concentrate on a single ethnic group. However, the diverse set of characteristic features observed in cephalometric analyses for patients from specific ethnic groups or populations residing in different regions necessitates a comprehensive comparative evaluation of individual indicators. This is crucial for accurately assessing the relationships between maxillary bases, alveolar processes, and teeth in both sagittal and vertical dimensions [13]. Given the recent introduction of a new parameter for assessing sagittal discrepancy—Tau angle analysis [3], which its authors claimed to be more reliable compared to ANB analysis—we aimed to conduct a comparative evaluation of both ANB angle analysis and Tau angle analysis in terms of assessing sagittal discrepancy. We also assessed the stability of identifying anthropometric points associated with each of these angles in relation to discrepancies between horizontal and vertical axes. Additionally, we conducted an analysis of the repeatability and reproducibility (R&R) of measurements, comparing the variability in landmark identification by the same observer and different observers in the same study. Furthermore, we evaluated the accuracy of classifying patients into one of three skeletal classes by analyzing both ANB and Tau angle values. Recent research has demonstrated the potential of employing artificial intelligence for precise and consistent analysis, even in 3D images. The outcomes of testing deep learning techniques based on Convolutional Neural Networks (CNNs) have shown them to be effective and accurate, comparable to the expertise of an experienced observer while being significantly faster. These findings suggest the feasibility of utilizing AI in orthodontic diagnostics, which holds substantial clinical importance in terms of result consistency and rapidity of analysis. It is worth noting, though, that the application of this technology to 3D images has limitations due to patient radiological protection considerations and should be reserved for the most complex cases. For simpler cases analyzed in 2D projection, this approach can be readily implemented [14,15].

Studies conducted by Maheen Ahmed, Attiya Shaikh, Mubassar, and Fida [11] regarding various cephalometric analyses have consistently shown that the ANB angle is the most precise and reliable indicator for evaluating the maxillomandibular relationship in the sagittal plane. The findings of our study support this conclusion as well. The ANB angle measurements exhibited smaller errors compared to the Tau angle measurements, primarily due to the narrower dispersion of horizontal coordinates of the points that have the greatest impact on angle measurement errors. The results for ANB measurements, including repeatability (1.61%), reproducibility (0.92% between different observers), interpatient variability (97.47%), and combined reproducibility and repeatability (2.53%), underscore the ANB angle’s dependency on individual variability. This emphasizes the ANB angle’s effectiveness as a standard parameter for evaluating sagittal relationships. The reliability of our data was further enhanced by the double-blinding of the study sample, where observers were unaware of both the clinical parameters of patients and the purpose of the analyses, ensuring unbiased evaluation.

Regarding errors associated with the identification of anthropometric points [10,12,16,17,18,19,20,21,22], the majority of studies that have evaluated the reproducibility and reliability [1,3,10,12,20,21,22,23,24,25,26] of individual landmarks have reported errors in point identification. Accurate assessment and repeatability are particularly crucial in growing patients, especially when dealing with the imprecise determination of points A, N, and B. These points can undergo significant positional changes during growth and orthodontic treatment, making it challenging to accurately assess the sagittal discrepancy between the jaws [25]. The reliability of anthropometric point determination is a critically important factor that underpins the repeatability of both linear and angular measurements [1,3,10,12,20,21,22,23]. When the identification of points cannot be consistently and reliably reproduced, the overall measurement used in cephalometric analysis becomes susceptible to significant errors that can impact diagnosis and treatment planning. In the sagittal analysis of the ANB angle, the precise evaluation of points A and B in relation to the x-axis, as well as the evaluation of point N in relation to the y-axis, holds particular significance [12]. Similarly, in the assessment of the Tau angle, attention should be paid to the accurate evaluation of points T and M in relation to the x-axis and point G in relation to the y-axis. The findings from our study suggest that the discrepancy along the horizontal axis (x-axis) when determining points A and B is minimal, potentially indicating a high level of accuracy in influencing ANB angle measurements. In a study by Durao [24], the ICC for the x-component at point B was less than 0.9, and for the y-axis at point N, it was also less than 0.9. However, the ICC values consistently ranged between 0.75 and 0.9, which was considered good. The assessment of the vertical divergence of point N, which is significantly higher compared to that of its horizontal component, aligns with this observation. Moreover, the intragroup correlation coefficients demonstrate high consistency in point identification, indicating the measurement’s high reliability when assessed by different observers. Similar results were obtained in the study conducted by Durao et al. [24].

The introduction of a new cephalometric measurement has the potential to significantly reduce errors in ANB measurement, particularly in growing patients who frequently undergo changes in the positions of points A, N, and B. Nevertheless, the adoption of this new measurement should also be approached with a degree of caution and careful consideration. Contrary to the claims made by Gupta P. et al. [3], it is evident from the present study that points M and G, while located near the central positions in the maxilla and mandibular symphysis, are not less susceptible to change compared to other points, including A and B. Furthermore, there is no clear evidence to support the assertion that the Tau angle is immune to mandibular rotation or consistently yields stable results for determining the correct direction of sagittal defects. The study also highlights that errors related to anthropometric point identification are more significant than errors in repeatability or reproducibility of landmarks in both ANB and Tau angle assessments. In terms of the ability to accurately classify patients into one of the three skeletal classes based on ANB and Tau angle measurements, the assessment remains similar, with the ANB angle continuing to serve as the gold standard.

The primary limitations of this study were the number of patients and its cross-sectional nature, accompanied by associated limitations. Other constraints encompassed alterations in the position of anthropometric points A, N, and B, and the potential for changes in points G and M due to the patients’ growth during puberty. Additionally, the accuracy of determining the T point presented a limitation. Consequently, further research is warranted, particularly randomized controlled trials (RCTs) comparing alternative methods for evaluating sagittal discrepancies in adequately large groups.

## 5. Conclusions

This study demonstrated significantly greater accuracy in measuring the ANB angle compared to the Tau angle. The Dahlberg error associated with the measurement of the Tau angle was approximately three times larger than that of the ANB angle. Furthermore, the intraclass correlation coefficient for the Tau angle was more than three and a half times smaller than that for the ANB angle. Similarly, the R&R error for the Tau angle measurement exceeded that for the ANB angle by more than threefold. In conclusion, the measurement results for the ANB angle exhibit lower levels of error when compared to the results for the Tau angle. Despite the introduction of new measurements based on novel landmarks, ANB angle analysis, considered the gold standard in assessing sagittal maxillomandibular relationships, remains a fundamental indicator for determining the sagittal direction of malocclusions.

## Figures and Tables

**Table 1:** Mean and standard deviation values of the ANB and Tau angles.

Patient	Class	ANB (°) M ± SD	Tau (°) M ± SD
A	I	2.2 ± 0.4	33.0 ± 1.5
B	II	5.7 ± 1.0	40.4 ± 2.2
C	III	0.2 ± 1.9	27.7 ± 4.2
D	I	4.7 ± 1.1	33.1 ± 2.5
E	I	1.3 ± 1.0	34.3 ± 2.0
F	II	7.1 ± 2.4	38.6 ± 3.5
G	II	5.3 ± 1.5	37.6 ± 2.7
H	III	−1.8 ± 1.6	25.8 ± 2.5
I	III	−0.7 ± 0.6	26.2 ± 2.2

The first digit is the average value, and the +/− sign is followed by the standard deviation (M ± SD—mean and standard deviation).

**Table 2:** Mean and standard deviation values of the ANB angle and coordinates of points A, N, and B measured on 9 cephalograms three times by 29 doctors (n = 87).

Patient	Class	ANB (°)	Point A		Point N		Point B	
			A_x_ (cm)	A_y_ (cm)	N_x_ (cm)	N_y_ (cm)	B_x_ (cm)	B_y_ (cm)
A	I	2.2 ± 0.4	5.99 ± 0.01	2.73 ± 0.04	5.97 ± 0.01	4.61 ± 0.02	5.89 ± 0.01	1.75 ± 0.03
B	II	5.7 ± 1.0	4.67 ± 0.02	2.87 ± 0.03	4.67 ± 0.01	4.56 ± 0.01	4.41 ± 0.02	1.99 ± 0.07
C	III	0.2 ± 1.9	5.04 ± 0.01	2.50 ± 0.05	5.00 ± 0.03	4.38 ± 0.08	5.08 ± 0.01	1.27 ± 0.04
D	I	4.7 ± 1.1	5.28 ± 0.04	3.29 ± 0.09	5.13 ± 0.01	5.20 ± 0.02	5.12 ± 0.01	1.93 ± 0.07
E	I	1.3 ± 1.0	5.92 ± 0.01	2.89 ± 0.04	5.96 ± 0.01	4.80 ± 0.02	5.83 ± 0.01	1.81 ± 0.04
F	II	7.1 ± 2.4	4.36 ± 0.02	2.77 ± 0.05	4.38 ± 0.01	4.52 ± 0.01	3.95 ± 0.01	1.64 ± 0.03
G	II	5.3 ± 1.5	5.90 ± 0.01	2.38 ± 0.04	5.96 ± 0.02	4.19 ± 0.08	5.61 ± 0.01	1.53 ± 0.06
H	III	−1.8 ± 1.6	7.29 ± 0.03	3.86 ± 0.08	7.65 ± 0.02	6.84 ± 0.03	7.24 ± 0.02	1.96 ± 0.12
I	III	−0.7 ± 0.6	4.70 ± 0.01	2.88 ± 0.04	4.80 ± 0.01	4.69 ± 0.01	4.67 ± 0.01	1.74 ± 0.04

The first digit is the average value, and the +/− sign is followed by the standard deviation (M ± SD—mean and standard deviation).

**Table 3:** Horizontal and vertical coordinate deviations of points A, N, and B measured three times by 29 doctors (n = 87) on 9 cephalograms, and 95% confidence intervals of the ANB angle.

Patient	ΔA_x_ (cm)	ΔA_y_ (cm)	ΔA_y_/ΔA_x_	ΔN_x_ (cm)	ΔN_y_ (cm)	ΔN_y_/N_x_	ΔB_x_ (cm)	ΔB_y_ (cm)	ΔB_y_/ΔB_x_	ANB (°) [95% CI]
A	0.06	0.22	3.4	0.06	0.17	2.6	0.04	0.14	3.4	[2.1; 2.3]
B	0.11	0.17	1.5	0.07	0.06	0.8	0.17	0.57	3.4	[5.6; 6.0]
C	0.06	0.24	3.8	0.37	0.45	1.2	0.04	0.19	4.2	[−0.4; −0.2]
D	0.19	0.38	2.0	0.08	0.18	2.2	0.07	0.36	5.3	[4.4; 5.1]
E	0.07	0.20	3.0	0.08	0.15	1.8	0.03	0.20	6.5	[1.1; 1.3]
F	0.10	0.24	2.4	0.05	0.07	1.3	0.06	0.20	3.4	[7.6; 8.0]
G	0.05	0.18	3.4	0.09	0.34	3.7	0.12	0.59	4.8	[5.4; 5.8]
H	0.20	0.46	2.3	0.10	0.22	2.2	0.21	1.09	5.2	[−2.3; −2.0]
I	0.08	0.20	2.6	0.08	0.05	0.6	0.04	0.20	4.6	[−0.9; −0.7]
All	0.10	0.25	2.7	0.11	0.19	1.8	0.09	0.31	4.5	[−0.7; 5.3]

ΔA_x_ = −A_x_^max^ − A_x_^min^; −ΔA_y_− = −A_y_^max^ − A_y_^min^; −ΔN_x_− = −N_x_^max^ − N_x_^min^; −ΔN_y_− = −N_y_^max^ − N_y_^min^; −ΔB_x_− = −B_x_^max^ − B_x_^min^; −ΔB_y_− = −B_y_^max^ − B_y_^min^.

**Table 4:** Correlation coefficients between coordinates of points A, N, and B and the ANB angle (n = 87).

Patient	Class	ANB (°)	Point A		Point N		Point B	
			A_X_	A_Y_	N_X_	N_Y_	B_X_	B_Y_
A	I	2.2 ± 0.4	0.295 *	−0.267 *	−0.410 **	−0.081	−0.223 *	0.104
B	II	5.7 ± 1.0	0.394 **	−0.137	0.312 *	0.061	−0.273 *	−0.207
C	III	0.2 ± 1.9	0.117	0.026	−0.013	0.088	0.134	−0.227 *
D	I	4.7 ± 1.1	0.672 ***	−0.538 ***	0.073	−0.274 *	0.070	−0.212 *
E	I	1.3 ± 1.0	0.524 ***	−0.322 *	0.037	−0.237 *	0.096	−0.154
F	II	7.1 ± 2.4	−0.014	−0.194	−0.295 *	0.039	0.119	0.313 *
G	II	5.3 ± 1.5	−0.186	0.127	−0.072	−0.018	−0.312 *	0.014
H	III	−1.8 ± 1.6	0.241 *	−0.241 *	0.202	0.074	−0.012	−0.195
I	III	−0.7 ± 0.6	0.350 *	−0.340 *	−0.085	−0.115	0.124	0.048

* *p* < 0.05, ** *p* < 0.001, *** *p* < 0.001.

**Table 5:** The results of multiple regression analysis for the ANB angle—model parameter estimates.

Patient	Class	Constant	Point A		Point N		Point B	
			A_x_ (cm)	A_y_ (cm)	N_x_ (cm)	N_y_ (cm)	B_x_ (cm)	B_y_ (cm)
A	I	72.5	13.44	-	−14.31	-	-	-
B	II	-	20.19	-	-	-	−13.72	-
C	III	-	13.53	-	-	-	-	-
D	I	-	17.12	-	-	-	−13.02	-
E	I	−179.7	38.56	-	-	−9.83	-	-
F	II	-	7.66	-	−81.68	-	-	-
G	II	349.51	-	-	-	-	−32.79	-
H	III	-	11.38	-	-	-	-	-
I	III	-	16.64	-	-	-	-	-

**Table 6:** Dahlberg error and error ratio for ANB angle (P).

Patient	Class		ANB	Point A		Point N		Point B	
				A_X_	A_Y_	N_X_	N_Y_	B_X_	B_Y_
A	I	Dahlberg error	0.286	0.008	0.016	0.009	0.020	0.004	0.021
		Error ratio	13.0%	0.1%	0.6%	0.1%	0.4%	0.1%	1.2%
B	II	Dahlberg error	0.330	0.010	0.027	0.007	0.006	0.008	0.039
		Error ratio	5.8%	0.2%	1.0%	0.1%	0.1%	0.2%	2.0%
C	III	Dahlberg error	0.345	0.009	0.023	0.040	0.059	0.006	0.038
		Error ratio	174.6%	0.2%	0.9%	0.8%	1.3%	0.1%	3.0%
D	I	Dahlberg error	0.665	0.024	0.036	0.009	0.016	0.010	0.040
		Error ratio	14.3%	0.5%	1.1%	0.2%	0.3%	0.2%	2.1%
E	I	Dahlberg error	0.265	0.008	0.022	0.011	0.016	0.006	0.029
		Error ratio	19.6%	0.1%	0.8%	0.2%	0.3%	0.1%	1.6%
F	II	Dahlberg error	0.371	0.011	0.016	0.007	0.008	0.007	0.024
		Error ratio	5.2%	0.2%	0.6%	0.2%	0.2%	0.2%	1.5%
G	II	Dahlberg error	0.338	0.007	0.020	0.008	0.061	0.004	0.029
		Error ratio	6.3%	0.1%	0.8%	0.1%	1.5%	0.1%	1.9%
H	III	Dahlberg error	0.435	0.023	0.040	0.012	0.016	0.025	0.123
		Error ratio	24.4%	0.3%	1.0%	0.2%	0.2%	0.3%	6.3%
I	III	Dahlberg error	0.291	0.007	0.018	0.008	0.008	0.007	0.032
		Error ratio	39.6%	0.1%	0.6%	0.2%	0.2%	0.2%	1.8%

**Table 7:** Intraclass correlation coefficient (ICC) values for the ANB.

	ANB (°)	A_x_ (cm)	A_y_ (cm)	N_x_ (cm)	N_y_ (cm)	B_x_ (cm)	B_y_ (cm)
ICC	0.841	0.999	0.985	1.000	0.997	1.000	0.962

**Table 8:** Results of the analysis of repeatability and reproducibility for the ANB angle measurement (°). R&R—Repeatability and Reproducibility).

Source of Variance	Estimated Sigma	Estimated Variance	R&R (%)	Total (%)
Repeatability (3 repetitions of measurements)	0.3733	0.1393	63.72	1.61
Reproducibility (29 orthodontists)	0.2816	0.0793	36.28	0.92
Patient (9 cephalograms)	2.9001	8.4108		97.47
Total R&R	0.4676	0.2187	100.00	2.53
Total	2.9376	8.6294		100.0

**Table 9:** Mean and standard deviation values of the Tau angle and coordinates of points T, G, and M measured on 9 cephalograms three times by 29 doctors (n = 87).

Patient	Class	Tau (°)	Point T		Point G		Point M	
			T_x_ (cm)	T_y_ (cm)	G_x_ (cm)	G_y_ (cm)	M_x_ (cm)	M_y_ (cm)
A	I	33.0 ± 1.5	3.85 ± 0.05	4.38 ± 0.03	5.76 ± 0.02	1.25 ± 0.03	5.81 ± 0.03	2.79 ± 0.02
B	II	40.4 ± 2.2	2.66 ± 0.01	4.46 ± 0.01	4.18 ± 0.02	1.61 ± 0.03	4.47 ± 0.04	2.89 ± 0.03
C	III	27.7 ± 4.2	2.85 ± 0.04	4.29 ± 0.03	4.93 ± 0.02	0.74 ± 0.04	4.82 ± 0.04	2.52 ± 0.03
D	I	33.1 ± 2.5	3.06 ± 0.05	4.80 ± 0.03	4.92 ± 0.02	1.50 ± 0.03	5.03 ± 0.05	3.35 ± 0.04
E	I	34.3 ± 2.0	3.55 ± 0.07	4.53 ± 0.03	5.70 ± 0.02	1.31 ± 0.04	5.71 ± 0.03	2.89 ± 0.02
F	II	38.6 ± 3.5	2.25 ± 0.05	4.41 ± 0.03	3.75 ± 0.02	1.21 ± 0.05	4.17 ± 0.04	2.82 ± 0.02
G	II	37.6 ± 2.7	3.89 ± 0.08	4.25 ± 0.03	5.44 ± 0.02	1.11 ± 0.03	5.72 ± 0.03	2.44 ± 0.03
H	III	25.8 ± 2.5	4.37 ± 0.05	6.71 ± 0.03	6.99 ± 0.02	1.27 ± 0.06	6.98 ± 0.07	3.95 ± 0.07
I	III	26.2 ± 2.2	2.79 ± 0.04	4.67 ± 0.03	4.58 ± 0.03	1.28 ± 0.04	4.52 ± 0.03	2.91 ± 0.03

The first digit is the average value, and the +/− sign is followed by the standard deviation (M ± SD—mean and standard deviation).

**Table 10:** Horizontal and vertical coordinate deviations of points T, G, and M measured three times by 29 doctors (n = 87) on 9 cephalograms, and 95% confidence intervals of the Tau angle.

Patient	ΔT_x_ (cm)	ΔT_y_ (cm)	ΔT_y_/ΔT_x_	ΔG_x_ (cm)	ΔG_y_ (cm)	ΔG_y_/G_x_	ΔM_x_ (cm)	ΔM_y_ (cm)	ΔM_y_/ΔM_x_	Tau (°) [95% CI]
A	0.18	0.12	0.7	0.11	0.18	1.6	0.19	0.16	0.9	[32.5; 33.4]
B	0.09	0.08	0.9	0.10	0.19	1.9	0.23	0.19	0.8	[40.2; 41.4]
C	0.18	0.16	0.9	0.12	0.17	1.4	0.20	0.17	0.8	[26.3; 27.0]
D	0.17	0.15	0.9	0.09	0.18	2.0	0.34	0.23	0.7	[32.1; 33.3]
E	0.21	0.15	0.7	0.11	0.22	2.0	0.18	0.14	0.8	[33.9; 34.7]
F	0.20	0.11	0.5	0.13	0.24	1.9	0.20	0.13	0.6	[38.9; 40.2]
G	0.29	0.16	0.6	0.10	0.17	1.7	0.24	0.24	1.0	[37.4; 38.8]
H	0.23	0.17	0.7	0.09	0.35	3.7	0.33	0.37	1.1	[25.0; 25.9]
I	0.14	0.10	0.8	0.16	0.16	1.0	0.22	0.24	1.1	[25.5; 26.3]
All	0.19	0.13	0.7	0.11	0.21	1.9	0.24	0.21	0.9	[24.0; 40.4]

ΔT_x_ = T_x_^max^ − T_x_^min^; ΔT_y_ = T_y_^max^ − T_y_^min^; ΔG_x_ = G_x_^max^ − G_x_^min^; ΔG_y_ = G_y_^max^ − G_y_^min^; ΔM_x_ = M_x_^max^ − M_x_^min^; ΔM_y_ = M_y_^max^ − M_y_^min.^

**Table 11:** Correlation coefficients between coordinates of points T, G, and M and the Tau angle (n = 87).

Patient	Class	Tau (°)	Point T		Point G		Point M	
			T_X_	T_Y_	G_X_	G_Y_	M_X_	M_Y_
A	I		−0.595 ***	−0.584 *	0.085	−0.247 *	0.444 ***	0.127
B	II		−0.216 *	−0.036	0.059	0.018	0.371 ***	0.139
C	III		−0.051	−0.083	−0.077	0.020	0.211	0.195
D	I		0.055	−0.148	−0.136	0.013	0.262 *	−0.075
E	I		−0.308 **	−0.220 *	−0.055	−0.086	0.270 *	0.306 **
F	II		−0.324 **	−0.234 *	0.151	0.355 **	−0.114	−0.011
G	II		−0.426 ***	−0.034	−0.092	0.282 **	−0.109	−0.068
H	III		−0.056	−0.120	−0.037	0.045	0.211 *	0.078
I	III		−0.228 *	−0.221 *	−0.375 ***	0.290 **	0.430 ***	−0.320 **

* *p* < 0.05, ** *p* < 0.001, *** *p* < 0.001.

**Table 12:** The multiple regression analysis results for the Tau angle—model parameter estimates.

Patient	Class	Constant	Point T		Point G		Point M	
			T_X_ (cm)	T_Y_ (cm)	G_X_ (cm)	G_Y_ (cm)	M_X_ (cm)	M_Y_ (cm)
A	I	−18.6	−16.48	-	-	-	19.81	-
B	II	−98.7	−37.73	-	-	-	20.67	-
C	III	2.7	-	-	-	-	26.85	-
D	I	115.1	-	-	-	-	12.47	-
E	I	−10.88	-	-	-	-	26.11	-
F	II	56.97	−21.23	-	-	-	24.38	-
G	II	67.3	−14.78	-	-	24.91	-	-
H	III	−25.6	-	-	-	-	7.37	-
I	III	24.8	-	-	−32.00	-	32.67	-

**Table 13:** Dahlberg error and error ratio (P).

Patient	Class		Tau	Point T		Point G		Point M	
				T_X_	T_Y_	G_X_	G_Y_	M_X_	M_Y_
A	I	Dahlberg error	0.891	0.033	0.023	0.015	0.017	0.020	0.021
		Error ratio	2.7%	0.9%	0.5%	0.3%	1.4%	0.3%	0.7%
B	II	Dahlberg error	1.348	0.012	0.008	0.017	0.017	0.028	0.027
		Error ratio	3.3%	0.4%	0.2%	0.4%	1.1%	0.6%	0.9%
C	III	Dahlberg error	1.015	0.012	0.011	0.023	0.027	0.017	0.020
		Error ratio	3.7%	0.4%	0.2%	0.5%	3.6%	0.4%	0.8%
D	I	Dahlberg error	1.197	0.036	0.024	0.013	0.020	0.026	0.028
		Error ratio	3.6%	1.2%	0.5%	0.3%	1.3%	0.5%	0.8%
E	I	Dahlberg error	0.954	0.039	0.019	0.019	0.024	0.010	0.009
		Error ratio	2.8%	1.1%	0.4%	0.3%	1.9%	0.2%	0.3%
F	II	Dahlberg error	1.170	0.023	0.015	0.017	0.033	0.019	0.017
		Error ratio	3.0%	1.0%	0.3%	0.5%	2.8%	0.4%	0.6%
G	II	Dahlberg error	1.407	0.038	0.013	0.012	0.019	0.019	0.029
		Error ratio	3.7%	1.0%	0.3%	0.2%	1.7%	0.3%	1.2%
H	III	Dahlberg error	1.639	0.022	0.016	0.011	0.035	0.048	0.050
		Error ratio	6.4%	0.5%	0.2%	0.2%	2.7%	0.7%	1.3%
I	III	Dahlberg error	1.228	0.016	0.013	0.018	0.022	0.023	0.026
		Error ratio	4.7%	0.6%	0.3%	0.4%	1.7%	0.5%	0.9%

**Table 14:** Intraclass correlation coefficient (ICC) values.

	Tau (°)	T_X_ (cm)	T_Y_ (cm)	G_X_ (cm)	G_Y_ (cm)	M_X_ (cm)	M_Y_ (cm)
ICC	0.147	0.586	0.517	0.364	0.624	0.562	0.376

**Table 15:** Results of the analysis of repeatability and reproducibility for the Tau angle measurement (°). R&R- Repeatability and Reproducibility).

Source of Variance	Estimated Sigma	Estimated Variance	R&R (%)	Total (%)
Repeatability (3 repetitions of measurements)	1.0273	1.0552	52.21	4.30
Reproducibility (29 orthodontists)	0.9828	0.9658	47.79	3.94
Patient (9 cephalograms)	4.7457	22.5214		91.76
Total R&R	1.4216	2.0211	100.00	8.24
Total	4.9540	24.5425		100.00

**Table 16:** Comparison between the Dahlberg error, intraclass correlation coefficient (ICC), and repeatability and reproducibility (R&R) of ANB and Tau measurements made by 29 orthodontists, as well as mean coordinate deviations of points that most strongly affect angle measurements.

Angle	Dahlberg Error	ICC	Total R&R	DCA (cm)
ANB	0.265–0.665	0.841–1.000	2.53%	ΔA_x_ = 0.10
Tau	0.891–1.639	0.147–0.624	8.24%	ΔM_x_ = 0.24
Tau/ANB	2.91	0.27	3.26	2.40

DCA—the average deviation of the coordinate that determines the accuracy of the angle measurement.

**Table 17:** The Cohen’s kappa values based on the ANB compared to the Tau angle.

(a)				
ANB (°)	Class			Chi-squared test
	I n = 261	II n = 261	III n = 261	
0°–4° (n = 224)	193 (73.9%) ^A^	6 (2.3%)	25 (9.6%)	χ^2^ = 988.2 df = 4 *p* < 0.001 (A vs. BC)
>4° (n = 324)	66 (25.3%)	248 (95.0%) ^B^	10 (3.8%)	
<0° (n = 235)	2 (0.8%)	7 (2.7%)	226 (86.6%) ^C^	
(b)				
Tau (°)	Class			Chi-squared test
	I n = 261	II n = 261	III n = 261	
28°–34° (n = 209)	169 (64.7%) ^A^	9 (3.4%)	31 (11.9%)	χ^2^ = 890.1 df = 4 *p* < 0.001 (A vs. BC)
>34° (n = 347)	90 (34.5%)	248 (95.0%) ^B^	9 (3.4%)	
<28° (n = 227)	2 (0.8%)	4 (1.5%)	221 (84.7%) ^C^	
(c)				
Tau (°)	ANB (°)			Chi-squared test
	0°–4° n = 224	> 4° n = 324	< 0° n = 235	
28°–34° (n = 209)	130 (58.0%) ^A^	50 (15.4%)	29 (12.3%)	χ^2^ = 748.2 df = 4 *p* < 0.001 (A vs. BC)
>34° (n = 347)	73 (32.6%)	274 (84.6%) ^B^	0 (0.0%)	
<28° (n = 227)	21 (9.4%)	0 (0.0%)	206 (87.7%) ^C^	

(A) Kappa = 0.778 [0.741; 0.815], SE = 0.036, N = 783; (B) Kappa = 0.722 [0.682; 0.763], SE = 0.036, N = 783; (C) Kappa = 0.662 [0.618; 0.706], SE = 0.036, N = 783.

## Data Availability

The raw data are available upon request from the corresponding author.
